# Novel scripts for improved annotation and selection of variants from whole exome sequencing in cancer research

**DOI:** 10.1016/j.mex.2015.03.003

**Published:** 2015-03-12

**Authors:** Marcus Celik Hansen, Line Nederby, Anne Roug, Palle Villesen, Eigil Kjeldsen, Charlotte Guldborg Nyvold, Peter Hokland

**Affiliations:** a Department of Hematology, Aarhus University Hospital, Aarhus, Denmark; b Bioinformatics Research Centre, Aarhus University, Denmark

**Keywords:** Extended variation annotation, Whole exome sequencing, Customized exome analysis, Mathematica, Variation and mutation annotation, Hematological malignancies

## Abstract

Sequencing the exome is quickly becoming the preferred method for discovering disease-inducing mutations. While obtaining data sets is a straightforward procedure, the subsequent analysis and interpretation of the data is a limiting step for clinical applications. Thus, while the initial mutation and variant calling can be performed by a bioinformatician or trained researcher, the output from robust packages such as MuTect and GATK is not directly informative for the general life scientists. In attempt to obviate this problem we have created complementary Wolfram scripts, which enable easy downstream annotation and selection, presented here in the perspective of hematological relevance. It also provides the researcher with the opportunity to extend the analysis by having a full-fledged programming and analysis environment of Mathematica at hand. In brief, post-processing is performed by:

• Mapping of germ line and somatic variants to coding regions, and defining variant sets within Mathematica.
• Processing of variants in variant effect predictor.
• Extended annotation, relevance scoring and defining focus areas through the provided functions.

## Graphical abstract

**Figure:**
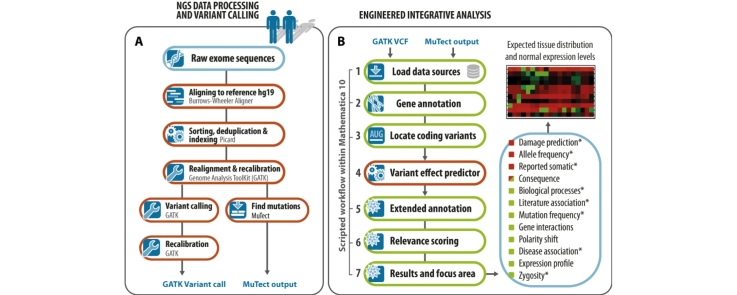

## Method

Here we present novel scripts to post-process called variants and mutations from whole exome sequencing. This simple method enables rapid evaluation of relevant and potentially disease contributing somatic or germ line coding single nucleotide variants (SNVs). It is a descriptive, integrative approach that can prove informative even in individual clinical cases. The method was developed in conjuction with exome analysis of a pair of monozygotic twins, and the following example is based on the processing of these data.

### (Part A) Whole exome data preprocessing and variant calling

Raw reads from sequenced purified T-cells (*control samples*) and mononuclear cells (MNC, *target samples*) were processed according to *GATK Best Practices* pre-processing workflow with default parameters, i.e., alignment to reference genome hg19 using the Burrows–Wheeler Aligner [1]. Sorting, removal of PCR duplicates and indexing was performed with Picard (Broad Institute, Cambridge, MA, US). Variant calling was based on GATK software package (Genome Analysis ToolKit Broad Institute, [2]) and somatic point mutations were detected by the MuTect software [3]. The biological samples were drawn from a pair of monozygotic twins with monoclonal B-cell lymphocytosis (Graphical abstract, part A. See the Additional information Section for more details).

### (Part B) Integrative analysis by extended variation annotation

This step requires Wolfram Mathematica (version 10, Wolfram Research, Oxfordshire, UK). Running the analysis on a modern workstation (8 GB RAM) will suffice. The latest public scripts, test sample and reference files can be downloaded at haematologi.dk/EVA. In this section we demonstrate analysis of MuTect and GATK called SNVs seen in the perspective of a hematological entity – here B-cell lymphocytic leukemia (B-CLL). As will be seen, this workflow is very simple to perform (Graphical abstract, part B. Steps with green bounding boxes represent scripted part of the workflow). All variable names are arbitrarily defined.

1) The functions are fetched and evaluated from the online resource by the following command:NotebookEvaluate[“http://haematologi.dk/EVA/scripts/EVA_0_1.nb”];Loading of reference data from UCSC genome annotation database [4] (UCSC, Santa Cruz, CA, USA), variant effect predictor [10], dbSNP [5], PubMed Catalogue of Somatic Mutations in Cancer (COSMIC, [6]), BioGPS [7], Uniprot and Entrez data (via Wolfram Research Server) and DisGeNET [8] is invoked next:LoadReferenceData[];

2) Variant mapping to RefSeq genes is performed with the GeneAnnotate function. Multiple *GATK Unified Genotyper* called sets can be annotated in a single procedure, as the following example shows:VariantsAnnotated = {GeneAnnotate[“controlsample1.vcf”], GeneAnnotate[“targetsample1.vcf”],GeneAnnotate[“controlsample2.vcf”], GeneAnnotate[“targetsample2.vcf”]};Single sets, for example somatic mutations detected by MuTect, can also be processed or combined with the same function as above. Processing of tabulated MuTect data requires an additional parameter “mutect”:MutationsAnnotated = GeneAnnotate[“mutations.tsv”,“mutect”];Because the UCSC gene reference matrix contains nearly fifty thousand entries, this step can be time-consuming, i.e., processing 35,000 SNVs took just over two minutes in our case.

3) Selecting coding SNVs is performed fast with the function FindCoding. This reduces the number of data entries substantially, and for standard exome sequencing this may be only focus of interest. There is no difference in processing MuTect detected mutations or GATK called variants:MutationsCoding = FindCoding[MutationsAnnotated[[1]]];Note that the first column of the matrix contains the mapped SNVs, the second (MutationsAnnotated[[2]]) stores the unmapped, i.e., intergenic variants. As before, sets can also be combined:VariantsAnnotatedCoding = {FindCoding[VariantsAnnotated[[1]]],FindCoding[VariantsAnnotated[[2]]],}Locating intersecting variants, i.e., to construct a pseudo-germline set is performed swiftly with the FindIntersection function (10^4^ SNVs in a few seconds). The newly constructed set can be stored in a variable:IntersectVariants = FindIntersection[VariantsAnnotatedCoding];Or saved as tabulated file with the filename *IntersectingVariants.tsv*:FindIntersection[VariantsAnnotatedCoding,“IntersectingVariants”];

4) The sets are returned in the Pileup format, which are then directly loaded into a local or online version of variant effect predictor (VEP), using the tab-separated values from previous. Make sure to select the proper version (e.g., Human GRCh37) and set VEP to return SIFT and PolyPhen scores and not predictions (see ensembl.org/info/docs/tools/vep for details). VEP input is neither restricted to coding regions nor is gene naming used in VEP. However, it is practical to narrow the sets for upload, referencing, gene search within Mathematica and, e.g., SNV quality analysis.

5) The results from VEP are imported into Mathematica, where the final annotation takes place. Disease focus – or foci – must be defined in order take full advantage of scoring and selection on the basis of disease entities. A MeSH-term is provided to find probable literature associations in combination with the respective genes (*Gene* AND “leukemia”[MeSH Terms]):diseasefocus = {“Myeloprolif”,“Leukemia”,“Lymphoma”,“Lymphocytosis”,“Myelodysplas”};MutVepAnno = EVA[“VEP_mutations.txt”,“leukemia”,diseasefocus];

6) The gathered information forms the basis of a rather crude, but efficient, scoring technique, based on global minor allele frequency (GMAF), damage prediction by Polyphen-2 and SIFT algorithms, literature search in the PubMed database, reported somatic mutations in COSMIC, current knowledge of functional role of the encoded protein, probable disorders associated with the aberrant gene and zygosity (Marked with asterisk in graphical abstract. The scoring contribution from literature search is, for example, based on the number of references in a logarithmic scale in order to dampen the effect of widely described biomarkers. See website haematologi.dk/EVA for latest details).MutVepAnnoSc = VarScore[MutVepAnno, funsrch, MutationsCoding];The coding variants (here the restricted set MutationsCoding from previous) are supplied in the last parameter of the function to combine the VEP result with GATK or MuTect called variants (i.e., information regarding zygosity and reads etc.). When searching for SNVs that are potentially relevant for the development or progression of cancer, it is practical to define a functional focus area. This must be done meticulously, but can have great impact in narrowing the scope. In hematological malignancies this search array can be defined as following, where the phrases reflects entries in Wolfram Research GenomeData:MutVepAnnoSc = {“SignalTransduction”, “CellAdhesion”, “SignalingPathway”, “Differentiation”, “CellProliferation”, “RegulationOfTranscription”, “Blood”, “Apoptosis”, “InflammatoryResponse”, “Immune”, “Chromatin”, “SignalingCascade”, “CellCycle”,“CellDivision”, “Mitosis”, “Hemopoiesis”, “B-cell”, “Methylation”, “Telomer”, “DNARepair”, “migration”, “kappa”, “SurfaceReceptor”, “T-cell”, “DefenceResponse”, “DNADamage”, “Phosporylation”};

7) Finally, result sets can be defined and retrieved and displayed with the function ResultTable:ResultTable[MutVepAnnoSc,MutationsCoding,funsrch,sift = 0.05,polyphen = 0.85,gmaf = 1,pubmed = 0,cosmic = 0,disassociation = 0,census = 0]Or simply justResultTable[MutVepAnnoSc,MutationsCoding,funsrch,0.05,0.85]

**Fig. 1:**
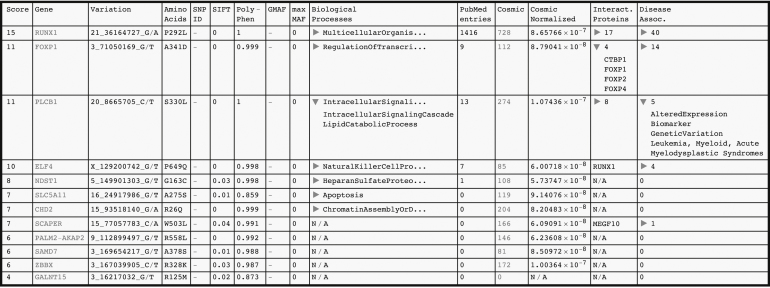
Representation of the interactive result table.

This returns SNVs defined in the search array, variants predicted to be damaged and with biological processes of the focus area. We could, however, have defined a threshold in the literature and disease association (e.g., pubmed = 1 and disassociation = 1) or return only COSMIC cancer census gene (census = 1), genes interacting with such (census = 2) or both (census = 3). Setting a maximum global minor allele frequency threshold is also trivial (e.g., gmaf = 0.01). A representation of the interactive result table is given in Fig. 1 based on mutations detected in one of the twins described, who had progressed to B-CLL, and the criteria/thresholds given above.

The SNVs are sorted by ranking score. Global minor allele frequencies (GMAF) are not present in this example, nor could a frequency be located in specific populations. This means that the mutations are likely unreported in the 1000 Genomes Project. Cosmic normalized is based on the number of COSMIC entries for a gene normalized to protein length. Online examples in the distributable computational document format (CDF), better suited for evaluation, are found on the public webpage haematologi.dk/EVA.

**Fig. 2:**
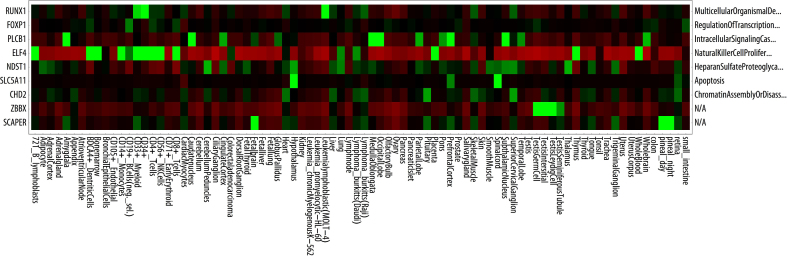
Pseudo-heatmap of the genes retrieved with result table with normalized data from BioGPS.

Getting a representation of the expected differential gene expression in various types of tissue can be practical when assessing the possible role of the genes. Thus, we provide the function HeatMap which displays a pseudo-heatmap of the genes called with ResultTable (if found in the array data reference). Please note that your data and this map only intersect by having the genes in common, and we do not attempt to provide anything else. From Fig. 2 it can be at least argued that the high ranked mutations are expressed by Hematopoietic progenitor cell antigen (CD34+) presenting cells, and thus may have a biological role, consistent with the literature. We have normalized the expression profiles, but data have been extracted through the BioGPS Dataset Library (from BioGPS.org).

The colors have been normalized to mean expression values. Visit BioGPS.org for an alternative representation. Note that bright green represents highest values, red lowest.

## Additional information

### Background

Exome sequencing provides a practical deep dive into some of the most important regions of the human genome, despite the fact that these constitute only a minor proportion of the latter. Based on the notion that this technology more rapidly enables identification of plausible causal genetic variants, it is attracting increasing attention. Its translation into clinical medicine, for the benefit of the single patient, seems imminent. Moreover, the price of whole exome, transcriptome, and genome sequencing will undoubtedly continue to drop, making large sequencing cancer studies more feasible. As a consequence from this, and information gained from deep sequencing, it will continue to change our understanding of the diverse biological fundament that, e.g*.*, drives cells towards malignancy.

Unfortunately, the easy access to massive sequence data output contrasts with the downstream analysis by the researcher, who will be the facilitator between the doctor and the patient. More specifically, how will the life scientist be able to manage the vast amount of raw data and quickly narrow it down to an informative list of variants and mutations, deciding what is relevant and what is not? Analysis tools, such as MuTect and GATK, initially process sequence alignment data in an efficient manner in terms of mutation and variant calling. However, the output formats returned, e.g*.* a large variant call format (VCF) file, can be bewildering. Likewise, the MuTect tabulated output is devoid of information on possible functional implications, whether the mutations are situated in coding or non-coding regions and what genes are implicated, presented in a readily readable format. Taken together, it can be a daunting and time-consuming task to reference such data sets and evaluate possible functional implications.

We encountered this problem when studying the possible germ line foundation underlying the susceptibility to monoclonal B-cell lymphocytosis and acquired mutations contributing to leukemic progression in monozygotic twins (submitted manuscript). To ease data interpretation we developed scripts, which enabled us to penetrate the otherwise bewildering amount of information and pinpoint possible contributors. Although we fully acknowledge that other informative annotation tools exists, such as ANNOVAR [9], SeattleSeq Annotation (NHLBI, Bethesda, MD, US) and exclusively commercial software such as the promising CLC Cancer Research Workbench (Qiagen Aarhus, DK), we hope that this workflow might help other researchers in evaluating the relevance of germ line variants and mutations in other neoplasms. The scripts are built to process the variants in a simple, accessible, and informative way, while keeping the option to take advantage of a full-fledged computing environment, when the need exists.

### Recapitulation of the developed method

The scripts were written in the Wolfram Language (version 10, Wolfram Research, Oxfordshire, UK) tied to external data sources in flat files. This means that no additional software installations are required. From the presented workflow (Graphical abstract) it will be seen that the scripts directly complement both GATK and Mutect in variant and mutation calling, as well as the first-line variant annotation tool variant effect predictor (VEP) [10]. The latest protocol, containing description on how to use the scripts, is found online at haematologi.dk/EVA. The method consists of two parts: (i) exome data pre-processing, in which GATK and MuTect output are selected and prepared for online VEP processing, and (ii) VEP post-processing by extensive gene annotation and relevance scoring. The motivation behind this divided approach is that while installing VEP on the local computer can be tedious, the maintained online version is fast, practical and free to use. Using the described research project as a working case the called single nucleotide variants are initially gene annotated (UCSC database, [4]) in order to select only variants mapped to RefSeq genes and coding variants (see Graphical abstract, part B).

Coding SNVs, or subsets of these, are subsequently converted to the pile-up format, feeding the VEP tool. In Ensembl’s VEP tool the variants are marked with, or restricted to, specific frequencies, Polyphen-2 [11] and SIFT [12] prediction scores etc. Importantly, the remainder of the annotation workflow is a straightforward task, as it requires only a few function calls after VEP output has been loaded into the scripts.

The second part of the workflow consists of: (i) gene annotation enrichment, (ii) scoring of variants based on automatically gathered information, and (iii) limiting output through a simple set of parameters, i.e*.* rare or unknown allele frequencies, damage prediction, functional implications etc. The latter is necessary to constrict the wealth of information, but merely represents an area of focus defined by the user. Apart from VEP derived information, the final output is enriched with polarity change, biological processes and interactions of the gene (through the Wolfram Research data servers), literature counts in the PubMed database, gene entries in the Cosmic database [8] normalized to protein length and disease association. The approach is based on a descriptive integrated evaluation and does not involve any statistical inference. This is reasonable and more informative when working with a low number of exome sequences, but these approaches are not mutually exclusive when analyzing sequence data; rather they are complementary.

## Concluding remarks

The hematological implications of the findings in our case are described elsewhere [13]. In short, we were able to suggest single nucleotide variants likely involved in B-cell proliferation. Clonal expansions of B lymphocytes is a area of hematology which deserves attention, since it is estimated that 1–3% of Caucasians over the age of 70 display such a feature. In addition, the germ line survey defined an area of focus that could help hypothesize models explaining inherited predisposition towards monoclonal lymphocytosis, and awaits screening in a larger cohort. In the described case several variants were rare and predicted to be damaged and can be potential contributors of the malignant process. Naturally, one cannot say, a priori, that a scrutinized variant of common frequency, not predicted to be damaged etc., does not contribute to the pattern of pathogenesis; nor can it be concluded that a high scoring, and probably damaged, variant does.

The Mathematica environment was chosen due to rapid development and prototyping, large library of functions and built-in communication with external data sources. We realized that, although consensus on how to process sequencing data is needed and strict uniformity is needed in clinical analyses, an important step in closing the gap between output data and meaningful results is to provide the life scientist with the right tools to assess the downstream data together with the knowledge of common pathways involved in diseases. The Mathematica environment and the developed scripts may provide such a platform and to train the researcher along the way – in a time where next generation sequencing is pushing forward. The support for formatted output and interactive reports is unprecedented, and along with the multi-paradigm programming style, this is one of the main reason to implement Mathematica here, e.g., in contrast to the like-wise versatile R. Care should always be taken when interpreting the impact of genome/exome data, but, in our opinion, with this method we were able to focus the wealth of information efficiently and rapidly get a clinically supporting picture in the described case. We hope to extend the work and, in the near future, to provide a free desktop application, where VEP processing is optional.
